# Diet, lifestyle factors and hypercholesterolemia in elderly men and women from Cyprus

**DOI:** 10.1186/1476-511X-4-17

**Published:** 2005-09-06

**Authors:** Evangelos Polychronopoulos, Demosthenes B Panagiotakos, Anna Polystipioti

**Affiliations:** 1 Department of Dietetics – Nutrition, Harokopio University, Athens, Greece

## Abstract

**Background:**

We sought to investigate the single and combined effect of Mediterranean diet, being physically active, moderate alcohol use, and non-smoking on clinical status of 150 elderly people from Cyprus.

**Methods:**

The study comprises individuals enrolled in surveys from Greece and Cyprus. This work includes 53 apparently men and 97 women, aged 65 to 100 years, from various areas of Cyprus. The cohort study was conducted between 2004 and 2005. A diet score that assesses the inherent characteristics of the Mediterranean diet was developed (range 0–55) and then a healthy index was calculated that evaluated four lifestyle habits (range 0 – 4), i.e. non-smoking, alcohol intake, physical activity and adherence to the Mediterranean diet (i.e. above the median of the score).

**Results:**

65% participants had hypercholesterolemia (total serum cholesterol > 200 mg/dl or use of lipid lowering agents). Moreover, 32% of the participants reported physically active, 5% reported smoking habits and 4% that they have stopped smoking during the past decade, while 8% reported alcohol drinking. A positive association was observed between prevalence of hypercholesterolemia and smoking habits (odds ratio = 4.3, p = 0.03), while an inverse association was observed between hypercholesterolemia, alcohol drinking (odds ratio = 0.3, p = 0.04) and adherence to a Mediterranean diet (odds ratio = 0.77, p = 0.02), controlled for age, sex, and other factors.

**Conclusion:**

Adherence to a Mediterranean diet and healthful lifestyle is associated with reduced odds of having hypercholesterolemia among elderly people.

## Introduction

During the past years several observational studies and clinical trials have revealed the adverse effect of abnormal blood lipid levels on the progression of atherosclerosis, and consequently the development of cardiovascular disease [1-5]. However, it should be noted that the strength of the relationships between blood lipids levels and atherosclerosis might be influenced by several lifestyle-related factors, like smoking, physical activity and psychosocial conditions. In particular, the Framingham Heart Study first reported that glucose intolerance, blood pressure levels and smoking habits modify the effect of total cholesterol on cardiovascular risk [1]. It is also well known that dietary patterns, like the Mediterranean diet, are strongly related with blood lipids levels, as well as with the prevalence and the management of dyslipidemia [6-10]. Recently, Yusuf et al. [11] reported that, among others, smoking, dietary habits and alcohol intake, as well as regular physical activity account for most of the risk of myocardial infarction worldwide in both sexes and at all ages in all regions. Moreover, in elderly people cardiovascular disease is the leading cause of death around the world, while hypercholesterolemia is among the major risk factors for the development of the disease. However, very few studies have evaluated the role of diet and other lifestyle-related factors in the prevalence of hypercholesterolemia in the elderly.

Given the lack of current data regarding the levels of blood lipids in Cyprus population, we investigated the distribution, awareness and management of high blood lipids levels, in a random sample of 65 years and older adults without any clinical evidence of cardiovascular disease. Moreover, we evaluated the association of dietary habits, smoking and physical activity with their blood lipid levels.

## Methods

### Population of the study

This study is a health and nutrition survey, which is being carried out in various areas of Cyprus (Arsos, Lemessos, Pachna, Pafos, Empa, Kallepia, Yeroskipou). From November 2004 to May 2005, 188 men and women, 65 years and older, and without any clinical history of cardiovascular disease were sere selected to participate in the study. Of them, 53 men and 97 women were finally agreed to participate (80% participation rate). People living in institutions were excluded from the study. The sampling was random and multistage according to the population of each city. All participants interviewed by specialists who used a standard questionnaire. The number of enrolled participants is adequate to evaluate standardised differences between the investigated parameters greater than 0.5, achieving statistical power >0.80 at <0.05 probability level (P-value).

In this work a special attention was given to people with hypercholesterolemia. In particular, hypercholesterolemia was defined as total serum cholesterol levels greater than 200 mg/dl or the use of lipid-lowering agents. Moreover, we recorded from participants' medical records high and low density lipoprotein (HDL, LDL) cholesterols, and triglycerides levels, as well as any special treatment.

### Dietary assessment

Consumption of 15 food groups was measured as an average per week during the past year through a semi-quantitative food-frequency questionnaire [12]. The frequency of consumption was then quantified approximately in terms of the number of times a month a food was consumed. Alcohol consumption was measured by daily ethanol intake, in wineglasses (100 ml and 12% ethanol concentration). Then we developed a dietary score according to the Mediterranean dietary pattern which consists of: (a) daily consumption: of non refined cereals and products (whole grain bread, pasta, brown rice, etc), vegetables (2 – 3 servings/day), fruits (6 servings/day), olive oil (as the main added lipid) and dairy products (1 – 2 servings/day), (b) weekly consumption: of fish (4 – 5 servings/week), poultry (3 – 4 servings/week), olives, pulses, and nuts (3 servings/week), potatoes, eggs and sweets (3 – 4 servings/week) and monthly consumption: of red meat and meat products (4 – 5 servings/month). In particular, for the consumption of food items that are close to this dietary pattern we assigned score 0 for rare or no consumption, 1 for 1 to 4 times/month, 2 for 5 to 8 times, 3 for 9 to 12 times/month, 4 for 13 to 18 times/month and 5 for almost daily consumption. On the other hand, for the consumption of foods that are away from this traditional diet, like meat and meat products, we assigned the opposite scores (i.e. 0 for almost daily consumption to 5 for rare or no consumption). Higher values of the suggested dietary score indicates adherence to the traditional Mediterranean diet (i.e. which is also characterized by moderate consumption of fat and high monounsaturated: saturated fat ratio).

### Lifestyle habits

To evaluate physical activity status of the patients during the past year we used a modified short version of a self-reported questionnaire, the International physical activity questionnaire (IPAQ) for the elderly [13]. Based on this questionnaire we assessed the frequency (times per week), duration (in minutes per time) and intensity of sports or occupation related physical activity. Participants who did not report any physical activities were defined as sedentary. For the rest of the participants we calculated a combined score by multiplying the weekly frequency, duration and intensity of physical activity. The upper tertile of the score classified participants as "highly" physical active, the medium tertile as "moderately" active and the lowest tertile as "low" physical active.

Finally, current smokers were defined as those who smoked at least one cigarette per day or have stopped cigarette smoking during the past 12 months. Former smokers were defined as those who had stopped smoking more than one year previously. The rest of them were defined as never smokers or rare smokers.

### The healthy index

To evaluate status of the participants we have developed a healthy index using dietary habits, smoking and physical activity status. In particular, participants who had diet score greater than the median (i.e. 36 for men and women) had a contribution to the healthy index score equal to 1, while the rest had a contribution equal to 0. Especially for alcoholic beverages intake we assigned in the healthy index the value of 1 for consumption of 3 or less wineglasses per day and the value of 0 for none or consumption of more than 3 wineglasses per day. Regarding the contribution of physical activity status in the healthy index we assigned the value of 1 for those who were in the middle or upper tertile of the physical activity score and the value of 0 in the rest of them. We have also assigned the value of 1 in the healthy index score into those who reported non-smokers or have stopped smoking for at least 15 years and the value of 0 into those who reported current or former smokers. Then we calculated the healthy lifestyle index by summing the individuals' scores for diet, alcohol intake, smoking and exercise. Thus, a 0 to 4 index was developed.

### Other measurements

The study's questionnaire also included demographic characteristics like age, gender, financial status (average annual income during the past three years) and education level. The educational level of the participants (as a proxy of social status) was measured by the years of school. Body mass index was calculated as weight (in kilograms) divided by standing height (in meters squared). Obesity was defined as body mass index > 29.9 Kg/m^2^. Participants who reported blood pressure levels were greater or equal to 140/90 mm Hg or were under antihypertensive medication were classified as hypertensives. Diabetes mellitus as a blood sugar > 125 mg/dl or the use of antidiabetic medication.

### Statistical analysis

Continuous variables are presented as mean values ± standard deviation. The categorical variables are presented as absolute and relative (%) frequencies. Associations between continuous variables and group of patients were evaluated through the analysis of variance (ANOVA), after controlling for equality of variances (homoscedacity). Due to multiple comparisons we applied the Bonferroni correction to correct for the inflation of Type-I error. Associations between categorical variables were tested by the use of the chi-squared test, without the correction of continuity. Correlations between continuous variables were tested by the use of Pearson's correlation coefficient. Multiple logistic regression analysis evaluated the association of the healthy index as well as individual scores on the likelihood of having hypercolesterolemia. All statistical calculations were performed on the SPSS version 12.0 software (SPSS Inc, Chicago, IL, U.S.A.).

## Results

Demographic, clinical and behavioural characteristics of the participants are presented in Table 1.

**Table 1:** Socio-demographic and lifestyle characteristics of the participants (% by gender)

	Men (n = 53)	Women (n = 97)	P
Age (years)	79 ± 8	75 ± 7	0.003
Education status (years of school)	5.7 ± 2	4.0 ± 2	0.001
Physical inactivity (%)	59	73	<0.001
Smoking habits (%)			<0.001
*Current smoker*	11	1	
*Former smoker*	47	1	
Never smoker	42	98	
Marital status (%)			<0.001
*Never married*		2	
*Married*	90	56	
*Divorced*		4	
*Widowed*	10	38	
Diet score (range 0 – 55)	36 ± 3	35 ± 3	0.15
Alcohol consumption (%)	15	4	0.02

Sixty five percent of the participants had hypercholesterolemia (60% of men and 68% of women, p = 0.34). Total serum cholesterol levels were 217 ± 38 mg/dl in men and 234 ± 42 mg/dl in women (p = 0.046). In addition to this information, 16% of men and 42% of women had cholesterol levels above 240 mg/dl (p < 0.001). Treatment of hypercholesterolemia was as follows: 37% of men and 53% of women were on special diet (p = 0.19), 81% of men and 63% of women on pharmaceutical treatment, i.e. statin and/or diet (p = 0.12) and 8% of men and 7% of women were untreated (p = 0.89). It is of interest that 90 out of 150 (60%) participants were unaware about the normal limits for total cholesterol levels. In addition, HDL cholesterol levels were 47 ± 9 mg/dl in men and 57 ± 14 mg/dl in women (p = 0.016), LDL cholesterol levels were 134 ± 19 mg/dl in men and 146 ± 32 mg/dl in women (p = 0.20) and triglycerides levels were 167 ± 75 mg/dl in men and 141 ± 62 mg/dl in women (p = 0.09). We also observed that 46% of men and 18% of women had low HDL-cholesterol levels (<40 mg/dl), 23% of men and 27% of women had high LDL-cholesterol levels (>100 mg/dl) and 52% of men and 40% of women had high triglycerides levels (>150 mg/dl). A group of people with particular interest is those who have normal total cholesterol, but low HDL cholesterol levels. In our population, 27% of men and women who had normal total cholesterol levels (i.e. <200 mg/dl) had HDL cholesterol levels lower than 35 and 45 mg/dl, respectively.

A positive association was observed between prevalence of hypercholesterolemia and smoking habits (p = 0.03), hypertension (p = 0.07), obesity (p = 0.06), while an inverse association was observed between hypercholesterolemia and alcohol drinking (p = 0.04). No associations were observed between hypercholesterolemia and financial status (p = 0.46), physical activity (p = 0.38) and presence of diabetes (p = 0.45).

It is known that age is a factor that correlates well with blood lipid levels. In our study, age was positively and significantly associated with all blood lipids measurements in both men and women. Thus further analysis confirmed previous findings between genders, after adjusting for age and controlling for other potential confounders that correlate with age and sex, like smoking habits, physical activity status and dietary habits (*results not shown in Tables or Figure*).

Since several investigators claimed for the effect of social status on blood lipid levels we evaluated the distribution of lipids in relation to years of school as a proxy of socio-economic level of the participants. After controlling for several potential confounders, like age, sex, body mass index, physical activity status, smoking and dietary habits, we found that none of the cholesterol levels had a consistent positive association with education status (*data not presented in text or Tables*). Moreover, no statistically significant associations were observed between blood lipids levels and income of the participants.

### Blood lipids and "healthy lifestyle status"

A secondary goal of this work was to assess blood lipid levels with dietary habits and other lifestyle characteristics. At first we evaluated the effect of Mediterranean diet on the investigated lipids. We revealed that greater adherence to Mediterranean diet (i.e. diet score > median value of 36) was associated with 23% lower likelihood of having hypercholesterolemia (odds ratio = 0.77, p = 0.02), after controlling for age, sex, body mass index, smoking habits and physical activity status. Afterwards we focused our interest on participants who were under lipid-lowering treatment and also adopted the Mediterranean diet. We found that the aforementioned combination was associated with 26% reduction in total serum cholesterol levels (p =< 0.001) and a 29% reduction in LDL cholesterol levels (p = 0.001), compared to those who were untreated and away to the Mediterranean diet. Of particular interest is that the observed reductions were higher than the reductions achieved by diet or statin treatment alone (p for interaction < 0.05). The effect of this combination on the other rest lipids was not statistically significant.

The association of the "healthy index" on the likelihood of having hypercholesterolemia is presented in Table 3. As we can see presence of 2 or more protective factors (i.e. healthy index > 1) seems to be associated with about 53% lower risk of having hypercholesterolemia. The calculation of the population attributable risk was showed that from 20% to 31% of hypercholesterolemic people could be prevented through this healthy lifestyle pattern. However, when we evaluated the components of the "healthy index" (Mediterranean diet, physical activity, moderate alcohol drinking and abstinence from smoking) on the prevalence of hypercholesterolemia no significant associations were observed. By the exception of Mediterranean diet mentioned above.

**Table 3:** Results from logistic regression analysis that evaluated the diet, physical activity, alcohol intake and smoking (healthy index) on the likelihood of having hypercholesterolemia

Healthy index	OR (95% CI)	PAR (%)	P
# *protective factors 0 (reference category)*	1.00		
*1*	0.33; 0.05 to 3.06	25	0.33
*2*	0.47; 0.22 to 0.97	20	0.01
*3*	0.20; 0.08 to 0.41	30	0.001
*4*	0.15; 0.07 to 0.29	31	0.001

OR = odds ratio; PAR = population attributable risk.

Variables also entered in the model were age, sex, body mass index, history of hypertension, and diabetes mellitus.

## Discussion

In this work we evaluated the distribution of blood lipid in a random, population-based, sample of elderly people from Cyprus. We observed that roughly six out of ten participants had high blood lipid levels. Although the latter finding may be influenced by the compliance to medication and other confounders, it is of great importance for the public health strategies in the studied population. Regarding the various dietary characteristics, we observed that adherence to the Mediterranean diet resulted a significant reduction on the likelihood of having hypercholesterolemia. In addition, we revealed the synergistic effect of Mediterranean diet with statin treatment in the management of blood lipids. Finally, a healthy lifestyle, including adherence to the Mediterranean diet, abstinence from smoking, physical activity, even in elderly people seems to be associated with a considerable reduction of the burden of hypercholesterolemia.

### Epidemiology of blood lipids among older adults

There are very few epidemiological studies that have assessed blood lipids levels in the elderly. Based on the ATTICA study [14] that evaluated, among others, blood lipids among Greek adults the investigators reported that 48% of men and 55% of women, aged > 50 years, had high total cholesterol levels (i.e. >200 mg/dl). In another Mediterranean population, the Portuguese, Costa et al. [15] reported that the prevalence of total cholesterol levels > 200 mg/dl among elderly people was 57%. In the EPICARDIAN study [16], the investigators reported that the prevalence of hypercholesterolemia among elderly Spanish people was 68%. Moreover, roughly 50% of white adult men and women in USA had total blood cholesterol levels over 200 mg/dl, as reported in the National Health and Nutrition Examination Survey (NHANES) III study [17]. These reports are in accord with our findings since we observed that 6 out of ten men and 7 out of ten women had high total serum cholesterol levels. Studies show that a higher percentage of women than men have total blood cholesterol of 200 mg/dl or higher, beginning at age 50 [1-3]. The later was confirmed by our study, too.

Regarding HDL cholesterol there is a wide range of scientific evidence which suggests that it plays a role in the development of coronary heart disease, especially in the elderly [1,3]. The NHANES III study [16] reported that 18% of middle aged men and 6% of middle aged women had HDL cholesterol levels below 35 mg/dl. In our study, 46% of men and 18% of women had low HDL-cholesterol levels. The increased rates may attribute to the increased age of our sample. Studies suggest that even for those with normal levels of total cholesterol, risk for myocardial infarction is high when HDL cholesterol is low. We observed that 27% of men and women, who had desirable total cholesterol levels, had low HDL cholesterol levels. The later may underline the importance of total-to-HDL cholesterol ratio for the evaluation of blood lipids and the prevention of atherosclerotic disease, at population level. According to several observational and clinical studies LDL cholesterol levels of 100 mg/dl or greater have a significant contribution for the development and the progression of coronary heart disease [1]. We observed that 23% of men and 27% of women had high LDL-cholesterol levels (>100 mg/dl). In the NHANES III these rates were 22% and 17%, respectively. Recently the Adult Treatment Plan (ATP) III [4] based initiation and treatment goals for dietary and pharmacological therapy on LDL cholesterol levels, number of pre-existing risk factors and previous experience of coronary heart disease. According to these guidelines individuals without coronary heart disease and less than two risk factors should initiate dietary therapy when LDL cholesterol levels exceed 160 mg/dl. Taking into account the high prevalence of the cardiovascular risk factors in our elderly population, it seems that a considerable proportion of men and women should be under lipid lowering agents. Finally, concerning triglycerides levels the ATP III suggests a cut off point of 150 mg/dl for defining elevated levels. In the present study, approximately one half of men and women had triglycerides levels of 150 mg/dl or higher. Unfortunately, population based data from other elderly cohorts regarding triglycerides levels are lacking; this makes the comparisons with our findings difficult. Nevertheless, it is noteworthy that a considerable proportion of men and women are at risk because of on their triglyceride levels.

Furthermore, we observed a positive association between prevalence of hypercholesterolemia and smoking habits, hypertension, and obesity, while no associations were observed between hypercholesterolemia and financial status, physical activity and presence of diabetes. The association between hypercholesterolemia with other co-morbidities has already been reported in other studies before [1-5]. This fact makes the presence of high blood levels of great importance for public health, and emerge measures for the management and control of hypercholesterolemia.

### Dietary and lifestyle management of blood lipids

It has already been reported that dietary habits usually influence blood lipids [7]. Thus, we evaluated lipid levels under the prism of the adoption or not of the Mediterranean diet. This diet has already been related with the reduction of all cause and cardiovascular disease mortality, due to its effect on blood pressure levels, body mass index, platelet aggregation, plasma fibrinogen and other haemostaseological factors [8-10]. Moreover, Petridou et al. [18] studying blood samples from Greek adolescents reported that the traditional Mediterranean pattern of living and eating was associated with a favorable lipid profile. However, benefits from this dietary pattern on blood lipids have rarely been reported in the literature, especially in the elderly. In our study we revealed that adherence to the Mediterranean diet is associated with a significant reduction in the likelihood of having hypercholesterolemia, after controlling for various potential confounders. Thus, based on this observation we could state another hypothesis of a pathophysiological mechanism by which Mediterranean diet may reduce cardiovascular risk, through the moderation of the oxidation process. Moreover, we revealed the additive effect of Mediterranean diet with statin treatment on blood lipids. Since the levels of uncontrolled or untreated dyslipidemia seem high in our population the previous finding could of high public health interest. However, the latter could be confounded by the better compliance to the treatment by people who were "closer" to the Mediterranean dietary pattern; which could not be assessed by the present study.

In addition, we observed an association between a "healthy index" and the likelihood of having hypercholesterolemia. In particular, presence of 2 or more protective factors (i.e. healthy index > 1) is associated with 53% lower risk of having hypercholesterolemia, while from 20% to 31% of hypercholesterolemic people could be prevented through this healthy lifestyle pattern. It is of interest that, by the exception of Mediterranean diet, none of the other components of the "healthy index" (physical activity, moderate alcohol drinking and abstinence from smoking) were associated with the prevalence of hypercholesterolemia in our elderly people.

#### Limitations

At this point it should be noted that the extrapolation of our findings into the general population might be under scrutiny. One of the main reasons is the moderate participation rate (68%), which is acceptable for population-based studies, like the present one, but may state hypotheses that the lifestyles of those who agreed to participate and of those who did not could be different. One other limitation is the cross-sectional design of the study. Thus, the observed benefits from the Mediterranean diet on oxidised LDL cholesterol levels, or the effect of physical activity on HDL cholesterol levels should be further investigated by randomised clinical trials.

## Conclusion

The present study revealed that a large proportion of elderly men and women have blood lipid abnormalities. Moreover, despite the aforementioned limitations, Mediterranean diet seems to be an effective, non-pharmacological, intervention for the management of high blood lipids levels. In addition, a healthy lifestyle that includes abstinence from smoking, and physical activity together with moderate alcohol drinking seems to be attractive in the prevention of hypercholesterolemia. Based on the likelihood that modification of lipid levels will be beneficial, especially, to the elderly that are at higher risk for coronary heart disease, we suggest that screening for these abnormalities is considered essential and must be followed by active and effective interventions.

**Table 2:** Clinical and anthropometric characteristics of the participants (% by gender)

	Men (n = 53)	Women (n = 97)	P
Hypertension (%)	60	58	0.85
Diabetes mellitus (%)	28	18	0.17
History of coronary heart disease (%)	11	5	0.20
Body mass index (kg/m^2^)	29 ± 4	30 ± 6	0.17
Obesity (%)	34	52	0.03
Waist circumference (cm)	107 ± 8	104 ± 8	0.11
Hip circumference (cm)	107 ± 7	113 ± 11	0.001
Waist to hip ratio	1.00 ± 0.06	0.92 ± 0.05	0.001
